# Effect of probiotics on nasal and intestinal microbiota in people with high exposure to particulate matter ≤ 2.5 μm (PM2.5): a randomized, double-blind, placebo-controlled clinical study

**DOI:** 10.1186/s13063-020-04759-4

**Published:** 2020-10-14

**Authors:** Yongcan Wu, Caixia Pei, Xiaomin Wang, Mingjie Wang, Demei Huang, Fei Wang, Wei Xiao, Zhenxing Wang

**Affiliations:** Hospital of Chengdu University of Traditional Chinese Medicine, No. 39 Shi-er-qiao Road, Chengdu, 610072 Sichuan Province People’s Republic of China

**Keywords:** Air pollution, Ambient particulate matter, Respiratory system, Microbiota, Probiotic, Randomized controlled trial

## Abstract

**Background:**

Extended exposure to high concentrations of PM2.5 changes the human microbiota profile, which in turn may increase morbidity and mortality due to respiratory system damage. A balanced microecosystem is crucial to human health, and certain health-related problems may be addressed by effective microecosystem regulation. Recent studies have confirmed that probiotics may reduce the incidence of respiratory diseases. However, few studies have investigated probiotic treatment outcomes in subjects exposed to high concentrations of PM2.5.

**Methods:**

This study is designed as a prospective, randomized, participants- and assessor-blinded, placebo-controlled trial. One hundred and twenty eligible volunteers recruited from October 2019 to July 2020 in downtown Chengdu, China, will be treated with either probiotics or placebo over 4 consecutive weeks. The primary outcome will be 16SrRNA sequencing assay data from nasal and intestinal secretions. Secondary outcomes will be pulmonary function, score on a gastrointestinal symptom rating scale, COOP/WONCA charts, and the Short-Form Health Survey 36 for quality of life. Results will be analyzed to assess differences in clinical efficacy between groups. Six-month follow-up examinations will evaluate the long-term value of probiotics on cardiovascular and respiratory disease end-point events.

**Discussion:**

We will explore the characteristics of nasal and intestinal microbiota in a population with high exposure to PM2.5. Probiotics and placebo interventions will be tested for efficacy in microbial balance regulation, effects on lung and physical functions, and quality of life improvement. This study is expected to provide reliable evidence to support the widespread promotion of probiotics in clinical practice for the protection of individuals with high exposure to PM2.5.

**Trial registration:**

Chinese Clinical Trial Registry ChiCTR1900025469. Registered on 27 August 2019.

## Background and rationale

Due to the rapid development of industrialization and urbanization in China, air pollution has become one of the main risk factors for diseases among Chinese citizens [1]. In particular, the recent and frequently occurring hazy weather caused by PM2.5 has seriously affected the health and quality of life of the population [2, 3]. PM (fine particulate matter) is the general name for solid and liquid particles suspended in ambient air [2, 4]. Particle size is the most important factor in air pollution, and PM2.5 is a fine particle with a size of ≤ 2.5 μm. PM2.5 may adsorb a variety of organic compounds, heavy metals, pathogenic microorganisms, and acid oxides and may enter the lower respiratory tract during respiratory movement, reaching the alveoli and possibly the blood circulation. These characteristics implicate PM2.5 as a potential cause of the recent increase in respiratory disease incidence [5]. Numerous epidemiological studies have revealed that long-term exposure to PM2.5 pose great risks to human health. Such as, the increase of PM2.5 concentration is closely correlated to the hospitalization rate and mortality associated with respiratory diseases [6–9]. Long-term exposure to PM2.5 can lead to an increase in emphysema [10] and small airway resistance [11]. The meta-analysis of global association of air pollution and heart failure indicated that air pollution especially the high concentration of PM2.5 has a close temporal association with heart failure hospitalization and heart failure mortality [12]. The studies also suggest that air pollution is one of the residual risk factors of CAD, and long-term exposure to fine particulate air pollution is associated with degree of coronary artery calcification, ischemic heart disease, and stroke mortality [13–15]. Another study shows that exposure to traffic air pollution may contribute to acute changes in blood pressure and autonomic and micro-vascular function in women [16]. High concentrations of environmental particles carry a large number of microorganisms, including many pathogens and opportunistic pathogens [17, 18]. Particulate matter is one of the key factors affecting the airborne bacterial concentration and community structure [19, 20]. Several studies demonstrated that high-levels of PM2.5/PM10 were related to alterations in the human pharyngeal [21], nasal [22], and intestinal [23] microbiota composition. Long-term exposure to PM2.5 as the main component in air pollution disrupts the inherent balance of the human microbiota and may induce and aggravate respiratory diseases. The nasal cavity as an independent official orifice, the nasal mucosa is not easily disturbed by diet, drugs, and other external factors, so the resident microbiota in the nasal cavity is also one of the typical characteristics of our body; it plays an important role in the maintenance of human health and the occurrence of diseases. Studies have confirmed the importance of nasal pathogens in lung diseases [24, 25]. It has been reported that exposure to environmental fine particles can also change the structure of nasal [26] and intestinal microbiota [27], so we choose nasal and intestinal microbiota as evaluation indicators.

The Food and Agriculture Organization of United Nations/World Health Organization (FAO/WHO) Expert Committee has defined probiotic strains as “live microorganisms which, when consumed in appropriate amounts in food, confer a health benefit on the host” [28]. The main probiotic component selected for this clinical trial is *Lactobacillus rhamnosus* GG (LGG). Studies confirmed that LGG may regulate the human microbiota, positively affects immunity, has anti-inflammatory properties, and produces a biofilm that can mechanically protect the mucosa [29]. However, few studies have investigated these positive effects on individuals exposed to high concentrations of PM2.5. A randomized, double-blind controlled clinical trial will be conducted in this study to investigate the characteristics of nasal and intestinal microbiota and the preventive and therapeutic effects of probiotics in healthy subjects exposed to an environment containing PM2.5. We hope to provide a clinical basis for the prevention and treatment of respiratory and circulatory diseases based on the study results.

## Methods

### Study design

**Fig. 1:**
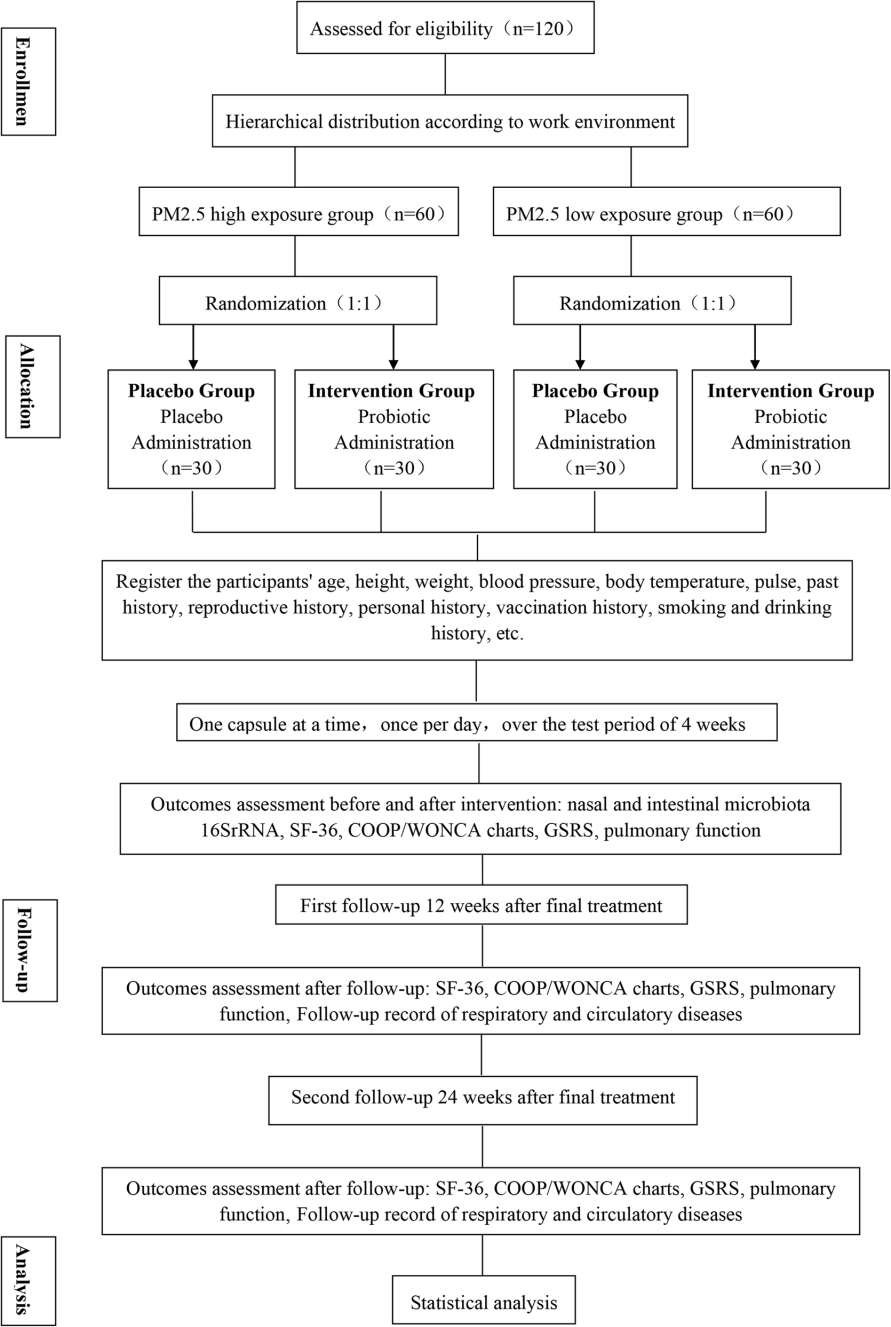
Study process: flowchart of study procedure

This study incorporates a randomized, double-blind, placebo-controlled clinical trial and was developed according to the Standard Protocol Items: Recommendations for Interventional Trials (SPIRIT) Statement (the SPIRIT checklist is shown in Additional file 1). The nasal and intestinal secretions of subjects exposed to PM2.5 will be collected and analyzed to determine the characteristics of nasal cavity and intestinal flora under high PM2.5 exposure and to investigate the clinical efficacy of probiotic intervention. A flowchart of this trial procedure is shown in Fig. 1. The Chinese Ethics Committee of Registering Clinical Trials (Ethical Review No.: ChiECRCT20190173) approved all the methods and ethics in the present study, and all procedures are in accordance with the Declaration of Helsinki v.08. Participants will sign a written informed consent form (see Additional file 2) with a clear understanding of the purpose, study procedure, and all potential risks related to the trial. All written informed consent forms will be collected and provided to members of the experimental group.

### Participant recruitment

We will recruit 120 candidates. The recruitment methods used in this study include (1) face-to-face communication, (2) bulletin boards, and (3) posters. We will post this recruitment information in neighborhoods and subdistrict offices based on the high concentration of PM2.5 published by the Environmental Supervision Administration, and the recruitment information will also be posted on the bulletin boards of the Environmental Protection Agency and the Transportation Administration. Members of the research group (WYC, WMJ, WXM, PCX, HDM) will be responsible for recruiting participants, all of whom are medical staff who have obtained physician certificates. After the start of the project, these members will receive the training of this project and take our project notice to communicate with the participants about the project. These trained members of the research group will obtain the written consent of the participants. Any eligible participant can contact our researchers using the provided telephone number and undergo screening to enter the group. Only those who fully meet the selection criteria, and sign the written informed consent form, will be registered and randomly assigned to placebo or probiotic treatment. All participants will be required to undergo a routine physical examination and complete the relevant questionnaire. There is no anticipated harm and compensation for trial participation. All subject personal information and data will be considered confidential. Only the members of our research group and the principal investigator will have access to the database. After the late data entry is completed, participants can log on to the website (http://www.medresman.org.cn/login.aspx) to query the details. The datasets analyzed during the current study are available from the corresponding author on reasonable request. Participant enrolment will begin in October 2019 and end in July 2020.

### Sample size

Sample size calculation was performed with G*Power 3.1 [30], using *F* test between factors with two groups (intervention and placebo) and three evaluation sessions (before administration, after administration, 12 and 24 weeks after administration). Since there was no pre-experiment before, we chose to take the average effect as 0.25, an *α* error probability of 0.05, and a power (1-β error probability) of 0.80. We take the Corr among rep measures as 0; the Nonsphericition correction ɛ is 1/(4–1) ≈ 1.34; the resulting total sample size is 95, because it has to be repeated 4 times; and the final sample size of each group is 24. Allowing for a maximum dropout rate of 30%, the number of subjects in the treatment group has been set to 30 participants. Based on the needs of our project, it is proposed to recruit high PM2.5 exposure and low PM2.5 exposure groups, and the groups are divided into intervention and placebo groups, according to each group requires a sample size of 30 patients, for a total of 120 patients.

### Selection criteria

#### Inclusion criteria for high-exposure population

We intend to include patients aged 22–65 with the following characteristics:

1. Male and female sanitation workers or traffic polices working in an environment with traffic pollution in Chengdu, for ≥ 3 years, and fixed working time ≥ 8 h
2. No history of gastroenteritis in the past month
3. No hormones or antibiotics were used for a period of 3 months before entering the study
4. No nasal spray or other probiotics (such as lactic acid bacteria, Bifidobacterium), yoghurt, or beverage used in the last 1 month
5. No lymph node enlargement, inflammation, polyps, mass, runny nose, etc., after clinical examination of lips, throat, ears, nostrils, and neck (especially anterior nostril)
6. No clinically important GI condition (e.g., inflammatory bowel disease, irritable bowel syndrome, gastric reflux, indigestion, dyspepsia, celiac disease, history of surgery for weight loss, gastroparesis, and clinically significant lactose and gluten intolerance or allergies) [31, 32]
7. No recent (within 2 weeks of visit 1; week − 1) history of an episode of acute GI illness such as nausea/vomiting or diarrhea (defined as ≥ 3 loose or liquid stools/day) [31, 32]

#### Inclusion criteria for low-exposure population

We intend to include patients aged 22–65 with the following characteristics:

1. Male and female staff of Chengdu Urban Environmental Hygiene Administration Bureau and Chengdu Traffic Administration Bureau, working indoors (with air conditioning), for ≥ 3 years, and working time ≥ 8 h
2. No history of gastroenteritis in the past month
3. No hormones or antibiotics were used for a period of 3 months before entering the study
4. No nasal spray or other probiotics (such as lactic acid bacteria, Bifidobacterium, etc.), yoghurt, or beverage used in the last one months;
5. No lymph node enlargement, inflammation, polyps, mass, runny nose, etc., after clinical examination of lips, throat, ears, nostrils, and neck (especially anterior nostril)
6. No clinically important GI condition (e.g., inflammatory bowel disease, irritable bowel syndrome, gastric reflux, indigestion, dyspepsia, celiac disease, history of surgery for weight loss, gastroparesis, and clinically significant lactose and gluten intolerance or allergies) [31, 32]
7. No recent (within 2 weeks of visit 1; week − 1) history of an episode of acute GI illness such as nausea/vomiting or diarrhea (defined as ≥ 3 loose or liquid stools/day) [31, 32]

### Exclusion criteria

#### Criteria of excluded participants

1. History of gastrointestinal surgery
2. Long history of drug use
3. History of smoking
4. Pregnancy and lactation
5. Could not understand and cooperate with the experimental process
6. Participated in other clinical trials within the past month

### Randomization and allocation concealment

Random numbers will be generated by BMISPSSStatistics24.0 software. We will include a total of 120 participants, including 60 participants in the high-exposure group and 60 participants in the low-exposure group. Firstly, the participants will be automatically assigned to the PM2.5 high-exposure group and the low-exposure group according to their work type and PM2.5 exposure time. Then given the number of seeds, 60 participants in the PM2.5 high-exposure group or the low exposure group will be randomly assigned to the intervention group and the placebo group. Professional statisticians will generate random allocation tables and grouping records. The random distribution table will be created in quadruplicate, one each by the principal investigator in charge of the project (WF), the project supervisor (WZX), the pharmacist (ZC), and the statistician (WZC).

### Blinding

During the course of the experiment, neither the participants nor the researchers will have the grouping information. The participants will be divided into a placebo group and an intervention group. According to the stratification of high and low exposures, the drug number, label, and packaging for each subject will be compiled from 001 to 060, respectively. The random numbers will be sealed in double opaque envelopes and managed by the researcher (LSQ) in charge of the blinding method. Each subject will be equipped with a corresponding emergency letter, and the serial and drug number on the cover and stationery will be confirmed consistent with the label on the drug package. The test name, subject serial number, the group that corresponds with the test, and the specific drugs used will be indicated in the letter. In case serious adverse events occur during the medical process, the envelope will be opened to break the blinding in an emergency and should be kept in reserve by the researcher.

### Trial procedure

#### Treatment providers

Four qualified clinical doctors of Traditional Chinese Medicine (TCM) with significant clinical experience (WYC, WMJ, WXM, PCX) will conduct a physical examination and pre-group assessment for the participants. All clinicians will complete a training session held by the principal investigator (WF) elaborating the procedure for recording the details of every treatment on individual case report forms (CRFs), follow up with participants, deal with adverse events, and obtain hands-on practical training.

### Treatment regimen

According to the working environment and the PM2.5 concentration of the working environment, the participants will be automatically divided into two groups: PM2.5 high-exposure group and PM2.5 low-exposure group. We will recruit a total of 120 participants, including 60 in the high-exposure group and 60 in the low-exposure group. Of the 60 participants in the high-exposure group, 30 will be given probiotics and another 30 will be given placebos. The same is true of 60 participants in the low-exposure group of PM2.5, who will be assigned 30 participants to take probiotics and another 30 to receive a placebo.

1. Intervention group: The intervention group will get probiotics. The same batch of Culturelle capsules provided by American i-Health (Cromwell, CT, USA) will be swallowed with warm water at < 36 °C, one tablet at a time, once per day, over the test period of 4 weeks. The follow-up period will be 24 weeks. As our drugs are live probiotics, in order to maintain better efficacy, we will advise patients to put our medicine in the 4 °C refrigerator.

2. Placebo group: The placebo group will get placebos, the same batch of placebo made by placebo Experimental Center, School of Pharmacy, Chengdu University of TCM. The placebo is made of starch without any efficacy or side effects and also will be swallowed with warm water at < 36 °C, one tablet at a time, once per day, over the test period of 4 weeks. The follow-up period will be 24 weeks. All drugs will be stored in a 4 °C refrigerator.

3. The intervention group and placebo group capsules will be packaged with the same label, each box containing a 4-week dose, a clearly visible label on each package stating “trial only”, and other information including name, dose, administration schedule, storage condition indication, expiration date, and manufacturer name. The reception, handling, storage, and distribution of drugs will be the responsibility of Dr. Zhang Chen, a pharmacist. In this subject, we chose placebo as the comparator. Because it is a kind of “simulated” drug, its physical properties such as appearance, size, color, dosage form, weight, taste, and smell are the same as those of the experimental drug, but it does not contain the active ingredients of the experimental drug and can reflect the “absolute” efficacy and safety of the tested drugs.

4. If the patient strongly requests to quit, the trial can be suspended. The drug in this project is a dietary supplement with controllable safety risks and no definite adverse reactions for the time being. However, if the patient has diarrhea or nausea, the trial can be suspended and adverse events can be reported. This project mainly observed the intestinal and nasal microbiota of the participants. Therefore, drugs that interfere with the microbiota, such as antibiotics and other probiotics, are prohibited, while implementing for the treatment of other diseases will not require alteration and continue for both trial arms.

### Data and sample collection

The research data will be collected and managed using the Chinese Clinical Research Public Management platform (Res Man). Res Man is an electronic data collection and management system that records the management process for clinical trials, subject baseline data recorded during the trial, result data, and other related data based on the Internet, and transmits all to the central database for preservation and management. The experimental data may only be accessed and operated by the research team. The principal investigator will have access to real-time data but cannot make any changes to it.

All data collection staff will be trained on managing research questionnaires and evaluating body measurements, in accordance with the standard research programs to ensure the high quality and consistency of the questionnaire. The quality of life evaluation scale Short-Form Health Survey 36 (SF-36) [33], COOP/WONCA charts [34], and gastrointestinal symptom rating scale (GSRS) will be completed before entering the group, after the final treatment, 12 weeks after the final treatment, and 24 weeks after the final treatment. The respiratory and circulatory disease follow-up records will be acquired 12 weeks after treatment and 24 weeks after final treatment.

The pulmonary function data will be collected by respiratory specialists to ensure consistency, and examinations will be performed before entering the group, after the final treatment, 12 weeks after the final treatment, and 24 weeks after the final treatment.

Before the participants join the intervention and placebo group, they will be trained on how to collect and transport samples. Nasal secretions will be collected face-to-face by members of the research group. The participants will receive a cryopreservation box about − 20 °C before the fresh feces are collected. After the samples have been collected and stored in the storage box, the members of the research group will be informed immediately to transport the samples to the laboratory.

The procedure for subject nasal specimen collection will include wiping the surface of nasal mucosa with sampling paper and immediately placing it in an aseptic cryopreservation tube. After the samples of the left and right nasal cavity are collected, they will be combined into one sample. After samples are collected, they will be sealed in a self-sealing bag, placed on dry ice, transported to the laboratory within 2 h, and stored at − 80 °C.

The subject intestinal samples will be collected by using the fecal collection bowl (to avoid urine mixing). Subsequently, the samples will be dipped with sterile cotton swabs and transferred to an aseptic freezing tube, frozen in liquid nitrogen for more than 4 h, and then transferred to − 80 °C for preservation.

On the consent form, participants will be asked if they agree to use of their data should they choose to withdraw from the trial. Participants will also be asked for permission for the research team to share relevant data with people from the Universities taking part in the research or from regulatory authorities, where relevant. This trial does involve collecting biological specimens for storage. All samples will be destructed after use.

### Outcome measures

The assessment included flora analysis of nasal and intestinal secretions and questionnaires from researchers (all Chinese versions). Assessments will be started before allocation at screening (T0, baseline), 4 weeks after the final treatment (T1, primary endpoint), 12 weeks after the final treatment (T2, secondary endpoint), and 24 weeks after the final treatment (T3, final endpoint). The timeframe of data collection and assessments is shown in Table 1 (the SPIRIT figure).

**Table 1:**
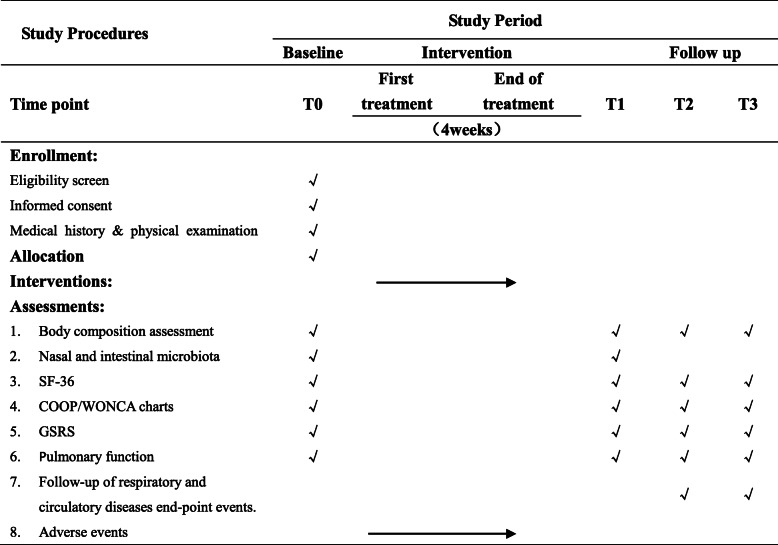
SPIRIT figure showing time points for enrollment, interventions and assessment

#### Primary outcome measure

The main primary outcomes in the current study will be nasal and gut microbiota diversity analyzed by the 16S ribosomal RNA (rRNA) gene, using Illumina Hiseq (Novogene Bioinformatics Technology Co., Ltd., Beijing, China). The differences in diversity in the PM2.5 high-exposure group and PM2.5 low-exposure group will be analyzed. We will also assess the changes between the treatment group and the placebo group after the intervention of probiotics in the same way.

#### Secondary outcome measures

Secondary outcome measures include the following:

1. The subject health will be assessed with a concise SF-36 questionnaire and a COOP/WONCA Chart will be generated. The subject quality of life will be summarized on eight aspects: physical function, role-physical, bodily pain, general health, vitality, social functioning, role-emotional, and mental health.
2. The gastrointestinal subject function will be evaluated with the score from the GSRS.
3. A pulmonary function test will be performed to evaluate the influence of PM2.5 on subject pulmonary function.
4. The long-term efficacy of probiotics will be evaluated by recording a follow-up table of cardiovascular (angina pectoris, arrhythmia, myocarditis, coronary heart disease, and heart failure) and respiratory diseases (respiratory infection, asthma, bronchitis, COPD) end-point events.
5. The participants’ weight, blood pressure, body temperature, pulse, past medical history, reproductive history, personal medical history, vaccination history, smoking history, and drinking history will also be registered to assess the basic health status of the patient.

### Adverse events’ reporting and safety monitoring

The Culturelle capsule used in this trial is a commercially available dietary supplement; therefore, the safety risk of the participants may be controlled, and the Chinese Ethics Committee of Registering Clinical Trials (Ethical Review No.: ChiECRCT20190173) has approved the study. We will ensure that every participant is aware of common adverse events related to the treatment in this study (such as indigestion or diarrhea), as well as serious adverse events (classified as life-threatening, permanently incapacitating, or requiring hospitalization) through the written consent form and detailed face-to-face consultations. During the treatment, participants must report any adverse events they may experience to the project team members, who must also record any adverse events they may have observed. All adverse events will be collected and presented promptly in detail to the project supervisor, who will determine the subsequent handling (including intimate observation, additional medical management, or early termination of participation) and record both the adverse events and the final outcomes in the CRF.

### Data management and monitoring

Data collection and monitoring will be managed by a specialized data and safety monitoring board (DSMB). The DSMB is composed of a statistician, a deputy chief physician in the respiratory department, and a junior Chinese medicine practitioner and will be established before the first enrolment of participants. The DSMB will be free to investigate all participant information and has no prior competitive interest with other members of the experimental group. The project team members will collect and record the original subject data with the CRFs, including a brief medical record, basic information, treatment records, pre- and post-treatment evaluation data, follow-up data, adverse event records, etc. Any changes to these paper-based data forms will not be allowed without the investigation and authorization of the DSMB. Two team members (PCX, WMJ) blinded to the allocation will responsible for copying information into a custom-designed and password-protected database on the Res Man Research Manager of the Clinical Trial Management Public Platform. Our data collection and management include paper case records and electronic data collection and management system. Paper case records will be reviewed and checked weekly by the principal investigator (WF). Electronic data collection and management can be carried out on the ResMan platform, which can carry out in-process quality control and online quality control of the data management process.

Missing data in clinical trials is inevitable, but it can be prevented as much as possible. Data management is the key to ensure the quality of data. In the process of dealing with missing data, we will choose different processing methods according to the judgment of data managers on the mechanism of missing data. For example, last observation carried forward, baseline observation carried forward, and worsts observation carried forward will be used to supplement the data. At the same time, it is necessary to make a sensitivity analysis of the test results. If the results of the sensitivity analysis are consistent with the results of the original analysis, it can be considered that the test conclusion has a strong credibility; on the contrary, if there is a great difference between the sensitivity analysis results and the original analysis results, it is necessary to further analyze the missing data to find the source of the difference.

### Adherence to study interventions

We will use several strategies to encourage and monitor compliance with research interventions. Clear oral and written instructions will be provided during the 7-month period from the beginning of the screening, to the end of the follow-up. The content will include guidance on lifestyle assessment and adherence to daily research capsule intake. Two weeks after the beginning of the study, all participants will be contacted to assess how they manage interventions and, if necessary, further personalized guidance will be provided. All unused medicines will be returned and documented.

### Statistical methods

Analysis will be performed using BMISPSSStatistics24.0 software. Results will be considered statistically significant if *p* ≤ 0.05. Since the data should compare changes within groups, between groups and between-groups, and in order to reduce the probability of errors, two-way analysis of variance of repeated measurements will be used to explore the impact of time and time-group effects, and Bonferroni post-test will be used to adjust. Analysis of variance (ANOVA) and Bonferroni post-processing analysis were used for comparison between-groups. Covariance analysis will be used to adjust the effects of confounding variables. Baseline descriptive data between the intervention and placebo group will be compared using chi-square for categorical variables and *t* tests for continuous variables. The primary outcome of intestinal and nasal microbiota will be performed with the R Programming Language 3.0.1 (NZL).

## Discussion

Exposure to PM2.5 can significantly increase the risk of respiratory and circulatory diseases. Clinical epidemiological evidence has demonstrated that symptoms of chronic bronchitis and abnormal pulmonary function are closely related to exposure to PM2.5 [35, 36]. Both short-term and long-term exposure to PM2.5 can directly lead to an increase in the incidence of respiratory diseases, clinical consultation rates, and hospitalization rates [37–39]. The microorganisms carried by PM2.5 induce the pro-inflammatory response of resident immune cells, increase intestinal permeability, and change the lumen environment of the intestine, leading to growth of specific microbial strains better suited for survival in an inflammatory environment. These changes in the microenvironment will alter the intestinal microbiota of the host [40–42].

Since few studies currently exist for reducing the toxicological effects of PM2.5, any additional effective interventions would be very valuable. It is accepted that the microecosystem plays a significant role in health, and interventions to modify the microbiota are demonstrating value for treatment of several conditions related to individual health. Probiotics form the basis of the interventions in this study and have the potential to address specific health concerns. The purpose of this study is to evaluate the protective effect of probiotics in subjects with high exposure to PM2.5 and to investigate possible microbial molecular mechanisms. The study results should provide valuable information regarding health maintenance and pharmaceutical intervention of probiotics in individuals with high exposure to PM2.5.

## Trial registration

The trial was pre-registered at the China Clinical Trials Registry on 27 August 2019 under the registration number ChiCTR1900025469. See http://www.chictr.org.cn/index.aspx

## Trial status

This paper is based on protocol version 2.0 dated 25 September 2019. Recruitment began in October 2019, and approximate date of completion is July 2020. Any major protocol changes will be notified to the ethics committee and updated on the Chinese Clinical Trial Registry.

## Modification of the protocol

Any changes to the protocol may affect the process of the study, which will be agreed between the project leader and the supervisor, inform the sponsors and all members of the research group, and also need to be approved by the Ethics Committee; any deviations of the protocol will be fully documented using a breach report form.

## Supplementary information

**Additional file 1.** The SPIRIT Checklist.

**Additional file 2.** Informed consent materials.

## Data Availability

Data sharing is not applicable to this article as no datasets are reported. Availability of datasets generated in the study will be included in papers reporting study outcomes. Access to the full protocol and model consent forms may be available from the author upon reasonable request.

## Abbreviations

CRF: Case report forms
DSMB: Data and safety monitoring board
FAO: Food and Agriculture Organization
GSRS: Gastrointestinal symptom rating scale
LGG: *Lactobacillus rhamnosus* GG
PM2.5: Fine particulate matter that have a diameter of less than 2.5 μm
Res Man: Research Public Management platform
SF-36: Short-Form Health Survey 36
SPIRIT: Standard Protocol Items: Recommendations for Interventional Trials
TCM: Traditional Chinese Medicine
WHO: World Health Organization
